# Crystal structure of 2-{[5-amino-1-(phenyl­sulfon­yl)-1*H*-pyrazol-3-yl]­oxy}-1-(4-methyl­phen­yl)ethan-1-one

**DOI:** 10.1107/S2056989021010008

**Published:** 2021-09-30

**Authors:** Nadia H. Metwally, Galal H. Elgemeie, Peter G. Jones

**Affiliations:** a Chemistry Department, Faculty of Science, Cairo University, Giza, Egypt; b Chemistry Department, Faculty of Science, Helwan University, Cairo, Egypt; c Institut für Anorganische und Analytische Chemie, Technische Universität Braunschweig, Hagenring 30, D-38106 Braunschweig, Germany

**Keywords:** pyrazole, sulfonyl­amino, hydrogen bond, crystal structure

## Abstract

In the title compound, C_18_H_17_N_3_O_4_S, the pyrazole ring is planar, with the sulfur atom lying 0.558 (1) Å out of the ring plane. The NH_2_ group is involved in an intra­molecular hydrogen bond to a sulfonyl oxygen atom; its other hydrogen atom forms an asymmetric three-centre hydrogen bond to the two oxygen atoms of the —O—CH_2_—C=O— grouping, *via* the 2_1_ screw axis, forming a ribbon structure parallel to the *b* axis. Translationally adjacent, coplanar ribbons form a layer parallel to (10).

## Abstract 2

In this *O*-alkyl­ated sulfonyl­pyrazolone, the sulfur atom lies 0.558 (1) Å out of the pyrazole ring plane. The NH_2_ group is involved in an intra­molecular hydrogen bond to a sulfonyl oxygen atom and in a three-centre system with the two oxygen atoms of the side chain at C3, forming a ribbon structure.

## Chemical context   

We are inter­ested in devising synthetic strategies for heterocyclic ring systems containing the *N*-sulfonyl- and *N*-sulf­onyl­amino moiety, which have shown significant biological activity as novel anti­viral and anti­microbial agents (Azzam *et al.*, 2017 ▸, 2019 ▸, 2020 ▸; Elgemeie *et al.*, 2017 ▸, 2019 ▸; Zhu *et al.*, 2013 ▸). In addition, some of our recently published *N*-aryl­sulfonyl­pyrazoles (Elgemeie & Hanfy, 1999 ▸; Elgemeie *et al.*, 1998 ▸, 2002 ▸, 2013 ▸) have been shown to be active as inhibitors of cathepsin B16 enzyme and NS2B-NS3 virus (Sidique *et al.*, 2009 ▸; Myers *et al.*, 2007 ▸). Based on these promising results, and in a continuation of our recent research to develop innovative and simple syntheses of other novel derivatives of *N*-sulfonyl­pyrazoles, we have begun to seek different scaffolds for use as potential pharmaceuticals (Zhang *et al.*, 2020 ▸). In particular, we have now synthesized an *O*-alkyl derivative of *N*-sulfonyl­amino­pyrazole **1**.

**Figure 1:**
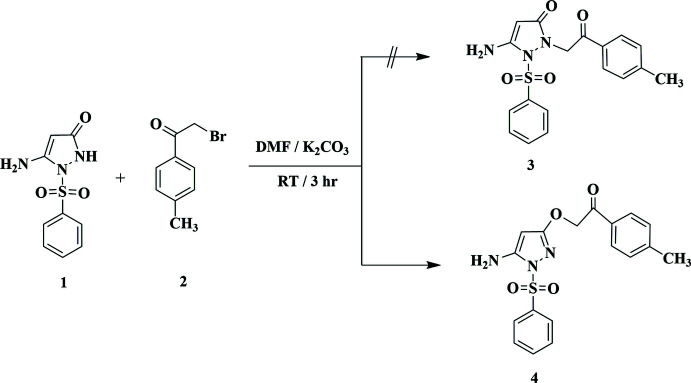
Reaction scheme for the preparation of the title compound **4**.

Thus, the reaction of 5-amino-1-(phenyl­sulfon­yl)-1,2-di­hydro-3*H*-pyrazol-3-one **1** with 2-bromo-1-(*p*-tol­yl)ethan-1-one **2** in *N*,*N*-di­methyl­formamide in the presence of potassium carbonate at room temperature furnished an adduct for which two possible isomers, the *O*-alkyl­ated or *N*-alkyl­ated *N*-sulfonyl­pyrazole structures (**3** or **4**) were considered. The ^1^H NMR spectrum of the product showed four singlet signals at δ = 2.40, 4.91, 5.45 and 6.34 ppm assigned for CH_3_, CH-pyrazole, CH_2_ and NH_2_ protons, in addition to signals assigned to aromatic protons. The available spectroscopic data cannot differentiate between structures **3** and **4** (Fig. 1 ▸). Thus, the X-ray structure of this product was determined, indicating unambiguously the formation of the *O*-alkyl­ated *N*-sulfonyl­pyrazole **4** as the sole product in the solid state.
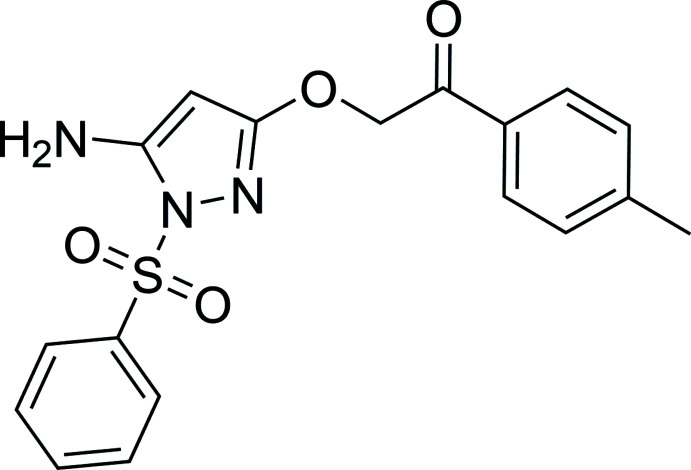


## Structural commentary   

**Figure 2:**
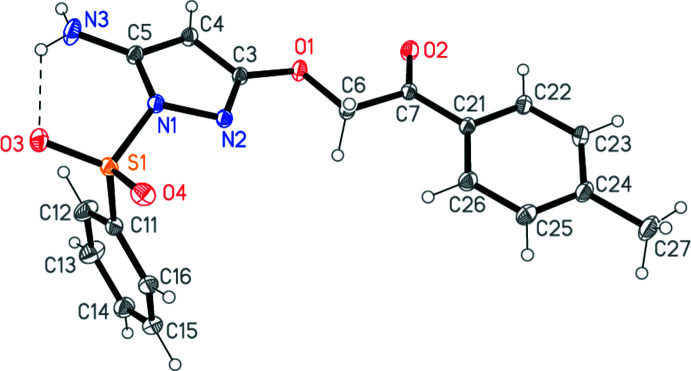
The mol­ecular structure of compound **4**. Ellipsoids represent 50% probability levels. The dashed line indicates an intra­molecular hydrogen bond.

The mol­ecular structure of **4** is shown in Fig. 2 ▸. Selected mol­ecular dimensions are given in Table 1 ▸. An intra­molecular hydrogen bond N3—H02⋯O3 is observed. The pyrazole ring is planar (r.m.s. deviation 0.015 Å) and its dimensions may be regarded as normal. The sulfur atom lies 0.558 (1) Å outside the ring plane, and the nitro­gen atom N1 is thus significantly pyramidalized; it lies 0.216 (1) Å out of the plane of the three atoms to which it binds. The atom sequence C4—C3—O1—C6—C7—C21—C22 presents an extended conformation, with all torsion angles close to ±180°. The planes of the pyrazole and the tolyl rings are thus almost parallel [inter­planar angle 14.46 (2)°].

## Supra­molecular features   

**Figure 3:**
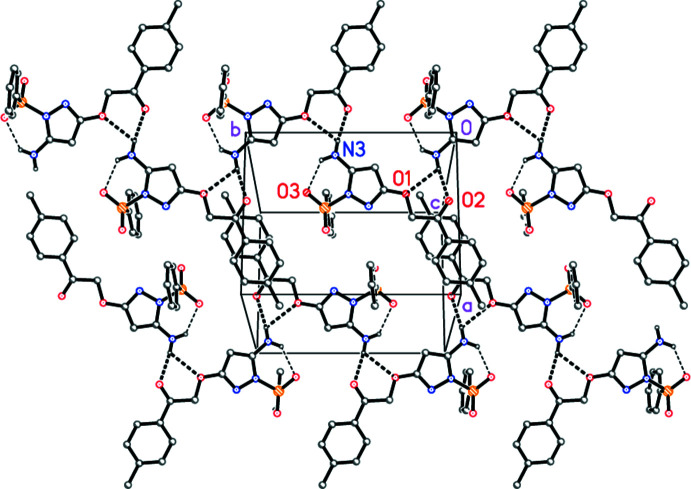
Packing diagram of compound **4** viewed perpendicular to (10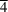) and centred on (1/2, 1/2, 1/2). Two ribbons parallel to the *b* axis are shown. Thick and thin dashed lines represent inter- and intra­molecular hydrogen bonds, respectively. Hydrogen atoms not involved in hydrogen bonding are omitted for clarity. Selected atoms of the asymmetric unit are labelled.

The classical hydrogen bond N3—H01⋯O2 (−*x*, *y* + 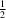, −*z* + 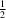) links the mol­ecules to form a broad ribbon structure parallel to the *b* axis. H01 also has a short but non-linear contact to O1 (same operator), representing the weaker component of an asymmetric three-centre system (Fig. 3 ▸). The vector between translationally adjacent, coplanar ribbons is [401], so that the layer of ribbons is parallel to (10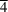). The second amine hydrogen atom H02 is only involved in the intra­molecular hydrogen bond (see above). The layers are linked by inter­actions C14—H14⋯O2 (*x*, −*y* + 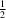, *z* + 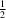), which connect every second layer, penetrating the layer in between. See Table 2 ▸ for details of hydrogen bonding.

## Database survey   

Version 5.41 of the Cambridge Structural Database (Groom *et al.*, 2016 ▸) was used for a CSD search with CONQUEST (Bruno *et al.*, 2002 ▸). The relative frequency of O- *vs* N2-alkyl­ation of such pyrazole ring systems was investigated by a search for pyrazoles with a C=O function at C3, H at C4, substituted at N2, no fused rings [as in our recent publication (Metwally *et al.*, 2021 ▸); 23 hits] or with substitution at the oxygen atom, H at C4, no substituent at N2, no fused rings (as here; 36 hits). Only one hit was registered for a pyrazole similar to **4** bearing a substitute at the C3—O group together with an N-substituent at C5 and an S-substituent at N1, namely 1-(4-fluoro­benzene­sulfon­yl)-5-amino-1*H*-pyrazol-3-yl thio­phene 2-carboxyl­ate, refcode YILPUF (Myers *et al.*, 2007 ▸).

## Synthesis and crystallization   

A mixture of 5-amino-1-phenyl­sulfonyl-1,2-di­hydro-3*H*-pyrazol-3-one **1** (0.01 mol), 2-bromo-1-(*p*-tol­yl)ethan-1-one **2** (0.01 mol) and anhydrous potassium carbonate (0.01 mol) in *N,N*-di­methyl­formamide (5 mL) was stirred at room temperature for 3 h. The mixture was poured onto ice–water; the solid that formed was filtered off and recrystallized from ethanol to give pale-brown crystals in 70% yield, m.p. 445 K. IR (KBr, cm^−1^): ν 3475, 3304 (NH_2_), 1690 (CO); ^1^H NMR (DMSO-d_6_): δ = 2.40 (*s*, 3H, CH_3_), 4.91 (*s*, 1H, CH pyrazole), 5.45 (*s*, 2H, CH_2_), 6.34 (*s*, 2H, NH_2_), 7.37 (*d*, 2H, *J* = 8.4 Hz, Ar), 7.56–7.60 (*m*, 2H, Ar), 7.72–7.76 (*m*, 3H, Ar), 7.85 (*d*, 2H, *J* = 8.0 Hz, Ar). Analysis calculated for C_18_H_17_N_3_O_4_S (371.41); C, 58.21; H, 4.61; N, 11.31; S, 8.63. Found: C, 58.39; H, 4.42; N, 11.65; S, 8.45%.

## Refinement   

Crystal data, data collection and structure refinement details are summarized in Table 3 ▸. The hydrogen atoms of the NH_2_ group were refined freely. The methyl group was refined as an idealized rigid group allowed to rotate but not tip, with C—H = 0.98 Å and H—C—H = 109.5°. Other hydrogens were included using a riding model starting from calculated positions (C—H_aromatic_ = 0.95, C—H_methyl­ene_ = 0.99 Å). The *U*(H) values were fixed at 1.5 or 1.2 times the equivalent *U*_iso_ value of the parent carbon atoms for methyl and non-methyl hydrogens, respectively. Six reflections were omitted because their calculated and measured *F*_o_^2^ and *F*_c_^2^ values differed by more than 7 s.u. The occurrence of such apparent outliers seems to be a general consequence of collecting data to high 2θ values (here 76°), whereby spherical atom scattering factors become less applicable. Special refinements using aspherical atom scattering factors can lead to greatly improved *R* values and thus fewer outliers, but this method is not yet widely employed. However, even for ‘normal’ refinement, it is still considered best practice to collect data to high diffraction angles wherever possible (Sanjuan-Szklarz *et al.*, 2016 ▸).

## Supplementary Material

Crystal structure: contains datablock(s) I, global. DOI: 10.1107/S2056989021010008/yz2011sup1.cif

Structure factors: contains datablock(s) I. DOI: 10.1107/S2056989021010008/yz2011Isup2.hkl

Supporting information file. DOI: 10.1107/S2056989021010008/yz2011Isup3.cml

CCDC reference: 2111897

Additional supporting information:  crystallographic information; 3D view; checkCIF report

## Figures and Tables

**Table 1:** Selected geometric parameters (Å, °)

N1—C5	1.3999 (7)	N2—C3	1.3141 (7)
N1—N2	1.4071 (7)	C3—C4	1.4167 (7)
N1—S1	1.6638 (5)	C4—C5	1.3758 (8)
			
C5—N1—N2	111.51 (4)	C5—C4—C3	104.42 (5)
C3—N2—N1	102.52 (4)	C4—C5—N1	106.35 (5)
N2—C3—C4	115.07 (5)	O4—S1—O3	119.98 (3)
			
O1—C6—C7—C21	179.72 (5)	C7—C6—O1—C3	−173.56 (5)
C4—C3—O1—C6	174.99 (5)	C6—C7—C21—C22	−173.11 (5)

**Table 2:** Hydrogen-bond geometry (Å, °)

*D*—H⋯*A*	*D*—H	H⋯*A*	*D*⋯*A*	*D*—H⋯*A*
N3—H01⋯O1^i^	0.869 (13)	2.497 (14)	3.0124 (7)	118.7 (11)
N3—H01⋯O2^i^	0.869 (13)	2.048 (13)	2.9121 (7)	172.8 (13)
N3—H02⋯O3	0.863 (12)	2.121 (13)	2.7806 (8)	132.8 (11)
C14—H14⋯O2^ii^	0.95	2.54	3.3458 (8)	142

Symmetry codes: (i) -x, y+{\script{1\over 2}}, -z+{\script{1\over 2}}; (ii) x, -y+{\script{1\over 2}}, z+{\script{1\over 2}}.

**Table 3:** Experimental details

Crystal data	
Chemical formula	C_18_H_17_N_3_O_4_S
*M*_r_	371.40
Crystal system, space group	Monoclinic, *P*2_1_/*c*
Temperature (K)	100
*a*, *b*, *c* (Å)	9.77236 (16), 11.98431 (18), 15.0131 (3)
β (°)	95.4487 (16)
*V* (Å^3^)	1750.32 (5)
*Z*	4
Radiation type	Mo *K*α
μ (mm^−1^)	0.21
Crystal size (mm)	0.3 × 0.2 × 0.1

Data collection	
Diffractometer	XtaLAB Synergy, HyPix
Absorption correction	Multi-scan (*CrysAlis PRO*; Rigaku OD, 2021 ▸)
*T*_min_, *T*_max_	0.917, 1.000
No. of measured, independent and observed [*I* > 2σ(*I*)] reflections	171365, 9400, 8324
*R*_int_	0.035
(sin θ/λ)_max_ (Å^−1^)	0.870

Refinement	
*R*[*F*^2^ > 2σ(*F* ^2^)], *wR*(*F* ^2^), *S*	0.031, 0.093, 1.06
No. of reflections	9400
No. of parameters	244
H-atom treatment	H atoms treated by a mixture of independent and constrained refinement
Δρ_max_, Δρ_min_ (e Å^−3^)	0.56, −0.43

Computer programs: *CrysAlis PRO* (Rigaku OD, 2021 ▸), *SHELXT* (Sheldrick, 2015*a* ▸), *SHELXL2018/3* (Sheldrick, 2015*b* ▸) and Siemens *XP* (Siemens, 1994 ▸).

## supplementary crystallographic information

### Crystal data

**Table:**

C_18_H_17_N_3_O_4_S	*F*(000) = 776
*M**_r_* = 371.40	*D*_x_ = 1.409 Mg m^−^^3^
Monoclinic, *P*2_1_/*c*	Mo *K*α radiation, λ = 0.71073 Å
*a* = 9.77236 (16) Å	Cell parameters from 110735 reflections
*b* = 11.98431 (18) Å	θ = 2.1–38.3°
*c* = 15.0131 (3) Å	µ = 0.21 mm^−^^1^
β = 95.4487 (16)°	*T* = 100 K
*V* = 1750.32 (5) Å^3^	Irregular, colourless
*Z* = 4	0.3 × 0.2 × 0.1 mm

### Data collection

**Table:**

XtaLAB Synergy, HyPix diffractometer	9400 independent reflections
Radiation source: micro-focus sealed X-ray tube	8324 reflections with *I* > 2σ(*I*)
Detector resolution: 10.0000 pixels mm^-1^	*R*_int_ = 0.035
ω scans	θ_max_ = 38.2°, θ_min_ = 2.1°
Absorption correction: multi-scan (CrysAlisPro; Rigaku OD, 2021)	*h* = −16→16
*T*_min_ = 0.917, *T*_max_ = 1.000	*k* = −20→20
171365 measured reflections	*l* = −26→25

### Refinement

**Table:**

Refinement on *F*^2^	Primary atom site location: dual
Least-squares matrix: full	Hydrogen site location: mixed
*R*[*F*^2^ > 2σ(*F*^2^)] = 0.031	H atoms treated by a mixture of independent and constrained refinement
*wR*(*F*^2^) = 0.093	*w* = 1/[σ^2^(*F*_o_^2^) + (0.0533*P*)^2^ + 0.2966*P*] where *P* = (*F*_o_^2^ + 2*F*_c_^2^)/3
*S* = 1.06	(Δ/σ)_max_ = 0.001
9400 reflections	Δρ_max_ = 0.56 e Å^−^^3^
244 parameters	Δρ_min_ = −0.43 e Å^−^^3^
0 restraints	

### Special details

**Table:**

Geometry. All esds (except the esd in the dihedral angle between two l.s. planes) are estimated using the full covariance matrix. The cell esds are taken into account individually in the estimation of esds in distances, angles and torsion angles; correlations between esds in cell parameters are only used when they are defined by crystal symmetry. An approximate (isotropic) treatment of cell esds is used for estimating esds involving l.s. planes. Least-squares planes (x,y,z in crystal coordinates) and deviations from them (* indicates atom used to define plane) - 4.1694 (0.0022) x - 1.2934 (0.0031) y + 14.0284 (0.0014) z = 3.1999 (0.0019) * 0.0038 (0.0004) C21 * 0.0012 (0.0004) C22 * -0.0045 (0.0004) C23 * 0.0029 (0.0004) C24 * 0.0021 (0.0005) C25 * -0.0054 (0.0004) C26 Rms deviation of fitted atoms = 0.0036 - 3.9412 (0.0025) x + 1.7088 (0.0035) y + 14.0838 (0.0015) z = 3.9392 (0.0017) Angle to previous plane (with approximate esd) = 14.458 ( 0.023 ) * -0.0213 (0.0003) N1 * 0.0180 (0.0003) N2 * -0.0081 (0.0003) C3 * -0.0053 (0.0003) C4 * 0.0167 (0.0003) C5 0.5584 (0.0008) S1 -0.0703 (0.0009) O1 -0.0102 (0.0010) N3 Rms deviation of fitted atoms = 0.0151 0.7595 (0.0030) x + 11.8725 (0.0006) y + 1.5620 (0.0042) z = 8.2715 (0.0020) Angle to previous plane (with approximate esd) = 77.814 ( 0.024 ) * -0.0025 (0.0004) C11 * 0.0002 (0.0005) C12 * 0.0021 (0.0006) C13 * -0.0021 (0.0005) C14 * -0.0001 (0.0005) C15 * 0.0025 (0.0004) C16 Rms deviation of fitted atoms = 0.0019

### Fractional atomic coordinates and isotropic or equivalent isotropic displacement parameters (Å^2^)

**Table:**

	*x*	*y*	*z*	*U*_iso_*/*U*_eq_	
N1	0.24056 (5)	0.51243 (4)	0.28333 (3)	0.01335 (7)	
N2	0.30323 (5)	0.41284 (4)	0.31574 (3)	0.01269 (7)	
C3	0.20639 (5)	0.33841 (4)	0.29582 (4)	0.01215 (8)	
C4	0.08065 (5)	0.38197 (4)	0.25555 (4)	0.01363 (8)	
H4	−0.001094	0.342184	0.236726	0.016*	
C5	0.10363 (5)	0.49489 (5)	0.24984 (4)	0.01353 (8)	
C6	0.36155 (5)	0.19759 (5)	0.34437 (4)	0.01441 (8)	
H6A	0.382782	0.227785	0.405500	0.017*	
H6B	0.429311	0.227836	0.305692	0.017*	
C7	0.36826 (5)	0.07135 (4)	0.34602 (4)	0.01344 (8)	
O1	0.22577 (4)	0.22853 (3)	0.31015 (3)	0.01574 (7)	
O2	0.26716 (5)	0.01561 (4)	0.32082 (4)	0.01960 (9)	
N3	0.02108 (6)	0.57726 (5)	0.21484 (5)	0.02257 (11)	
H01	−0.0666 (14)	0.5651 (12)	0.2040 (8)	0.036 (3)*	
H02	0.0528 (13)	0.6445 (10)	0.2171 (8)	0.028 (3)*	
S1	0.30852 (2)	0.62852 (2)	0.32942 (2)	0.01351 (4)	
O3	0.23014 (5)	0.71771 (4)	0.28591 (3)	0.01948 (8)	
O4	0.45358 (5)	0.62322 (4)	0.32464 (3)	0.01823 (8)	
C11	0.27468 (6)	0.62077 (5)	0.44193 (4)	0.01454 (9)	
C12	0.13852 (7)	0.62706 (6)	0.46205 (4)	0.02211 (12)	
H12	0.066048	0.637804	0.416031	0.027*	
C13	0.11104 (7)	0.61728 (7)	0.55093 (5)	0.02584 (13)	
H13	0.018996	0.621524	0.566106	0.031*	
C14	0.21846 (7)	0.60125 (6)	0.61789 (4)	0.02114 (11)	
H14	0.198893	0.594176	0.678417	0.025*	
C15	0.35380 (7)	0.59553 (5)	0.59684 (4)	0.01925 (10)	
H15	0.426314	0.584806	0.642829	0.023*	
C16	0.38275 (6)	0.60557 (5)	0.50806 (4)	0.01702 (9)	
H16	0.474871	0.602097	0.492941	0.020*	
C21	0.50072 (5)	0.01906 (4)	0.37895 (4)	0.01340 (8)	
C22	0.51276 (6)	−0.09678 (5)	0.37166 (4)	0.01651 (9)	
H22	0.437419	−0.139412	0.345499	0.020*	
C23	0.63438 (6)	−0.14961 (5)	0.40253 (4)	0.01829 (10)	
H23	0.641772	−0.228272	0.396829	0.022*	
C24	0.74621 (6)	−0.08873 (5)	0.44191 (4)	0.01787 (10)	
C25	0.73356 (6)	0.02713 (6)	0.44878 (4)	0.01941 (10)	
H25	0.808689	0.069659	0.475360	0.023*	
C26	0.61253 (6)	0.08085 (5)	0.41722 (4)	0.01695 (9)	
H26	0.605784	0.159693	0.421678	0.020*	
C27	0.87758 (7)	−0.14635 (7)	0.47616 (5)	0.02598 (13)	
H27A	0.865252	−0.227330	0.471113	0.039*	
H27B	0.901371	−0.126314	0.539016	0.039*	
H27C	0.951691	−0.122930	0.440676	0.039*	

### Atomic displacement parameters (Å^2^)

**Table:**

	*U*^11^	*U*^22^	*U*^33^	*U*^12^	*U*^13^	*U*^23^
N1	0.01168 (17)	0.01073 (16)	0.01710 (18)	−0.00019 (13)	−0.00153 (14)	−0.00017 (13)
N2	0.01097 (16)	0.01076 (16)	0.01599 (18)	0.00054 (13)	−0.00050 (13)	−0.00021 (13)
C3	0.01059 (18)	0.01128 (18)	0.01451 (19)	0.00047 (14)	0.00092 (14)	0.00002 (14)
C4	0.00988 (18)	0.01317 (19)	0.0175 (2)	−0.00025 (14)	−0.00052 (15)	0.00061 (15)
C5	0.01098 (18)	0.01337 (19)	0.0159 (2)	0.00071 (15)	−0.00055 (15)	0.00110 (15)
C6	0.01131 (18)	0.01194 (18)	0.0195 (2)	0.00104 (15)	−0.00122 (16)	−0.00021 (16)
C7	0.01256 (19)	0.01213 (18)	0.0153 (2)	0.00107 (15)	−0.00068 (15)	−0.00064 (15)
O1	0.01125 (15)	0.01075 (15)	0.02455 (19)	0.00083 (12)	−0.00184 (13)	0.00110 (13)
O2	0.01475 (18)	0.01379 (17)	0.0288 (2)	−0.00047 (13)	−0.00554 (15)	−0.00210 (15)
N3	0.0147 (2)	0.0154 (2)	0.0360 (3)	0.00168 (16)	−0.00609 (19)	0.00591 (19)
S1	0.01339 (6)	0.01073 (6)	0.01621 (6)	−0.00151 (4)	0.00040 (4)	0.00007 (4)
O3	0.0232 (2)	0.01187 (16)	0.0226 (2)	0.00004 (14)	−0.00214 (16)	0.00320 (14)
O4	0.01387 (17)	0.01884 (19)	0.0222 (2)	−0.00499 (14)	0.00286 (14)	−0.00164 (14)
C11	0.0134 (2)	0.01384 (19)	0.0161 (2)	0.00121 (15)	−0.00008 (16)	−0.00136 (15)
C12	0.0142 (2)	0.0340 (3)	0.0179 (2)	0.0062 (2)	0.00046 (18)	0.0004 (2)
C13	0.0176 (3)	0.0408 (4)	0.0194 (3)	0.0068 (2)	0.0032 (2)	0.0007 (2)
C14	0.0224 (3)	0.0240 (3)	0.0170 (2)	0.0040 (2)	0.00141 (19)	−0.00039 (19)
C15	0.0192 (2)	0.0198 (2)	0.0179 (2)	0.00189 (19)	−0.00336 (18)	−0.00113 (18)
C16	0.0139 (2)	0.0173 (2)	0.0192 (2)	0.00034 (17)	−0.00162 (17)	−0.00173 (17)
C21	0.01182 (19)	0.01317 (19)	0.0149 (2)	0.00123 (15)	−0.00019 (15)	0.00002 (15)
C22	0.0156 (2)	0.0138 (2)	0.0195 (2)	0.00256 (16)	−0.00126 (17)	−0.00044 (17)
C23	0.0175 (2)	0.0172 (2)	0.0199 (2)	0.00551 (18)	−0.00005 (18)	0.00038 (18)
C24	0.0140 (2)	0.0233 (3)	0.0162 (2)	0.00563 (18)	0.00076 (16)	0.00157 (18)
C25	0.0126 (2)	0.0226 (3)	0.0223 (3)	0.00085 (18)	−0.00198 (18)	−0.00033 (19)
C26	0.0131 (2)	0.0165 (2)	0.0208 (2)	0.00012 (16)	−0.00120 (17)	−0.00043 (18)
C27	0.0176 (3)	0.0355 (3)	0.0242 (3)	0.0113 (2)	−0.0016 (2)	0.0022 (3)

### Geometric parameters (Å, º)

**Table:**

N1—C5	1.3999 (7)	C22—C23	1.3869 (8)
N1—N2	1.4071 (7)	C23—C24	1.3976 (9)
N1—S1	1.6638 (5)	C24—C25	1.3987 (9)
N2—C3	1.3141 (7)	C24—C27	1.5047 (9)
C3—O1	1.3447 (7)	C25—C26	1.3898 (8)
C3—C4	1.4167 (7)	C4—H4	0.9500
C4—C5	1.3758 (8)	C6—H6A	0.9900
C5—N3	1.3494 (8)	C6—H6B	0.9900
C6—O1	1.4257 (7)	N3—H01	0.869 (13)
C6—C7	1.5144 (8)	N3—H02	0.863 (12)
C7—O2	1.2226 (7)	C12—H12	0.9500
C7—C21	1.4802 (8)	C13—H13	0.9500
S1—O4	1.4277 (5)	C14—H14	0.9500
S1—O3	1.4355 (5)	C15—H15	0.9500
S1—C11	1.7542 (6)	C16—H16	0.9500
C11—C16	1.3910 (8)	C22—H22	0.9500
C11—C12	1.3942 (9)	C23—H23	0.9500
C12—C13	1.3908 (10)	C25—H25	0.9500
C13—C14	1.3961 (10)	C26—H26	0.9500
C14—C15	1.3904 (10)	C27—H27A	0.9800
C15—C16	1.3938 (9)	C27—H27B	0.9800
C21—C26	1.3976 (8)	C27—H27C	0.9800
C21—C22	1.3984 (8)		
			
C5—N1—N2	111.51 (4)	C25—C24—C27	120.59 (6)
C5—N1—S1	127.24 (4)	C26—C25—C24	120.87 (6)
N2—N1—S1	114.96 (4)	C25—C26—C21	120.10 (6)
C3—N2—N1	102.52 (4)	C5—C4—H4	127.8
N2—C3—O1	122.74 (5)	C3—C4—H4	127.8
N2—C3—C4	115.07 (5)	O1—C6—H6A	110.2
O1—C3—C4	122.17 (5)	C7—C6—H6A	110.2
C5—C4—C3	104.42 (5)	O1—C6—H6B	110.2
N3—C5—C4	130.45 (5)	C7—C6—H6B	110.2
N3—C5—N1	123.07 (5)	H6A—C6—H6B	108.5
C4—C5—N1	106.35 (5)	C5—N3—H01	119.6 (9)
O1—C6—C7	107.65 (4)	C5—N3—H02	117.9 (8)
O2—C7—C21	121.83 (5)	H01—N3—H02	120.5 (12)
O2—C7—C6	120.55 (5)	C13—C12—H12	120.7
C21—C7—C6	117.62 (5)	C11—C12—H12	120.7
C3—O1—C6	115.11 (4)	C12—C13—H13	119.9
O4—S1—O3	119.98 (3)	C14—C13—H13	119.9
O4—S1—N1	107.51 (3)	C15—C14—H14	119.7
O3—S1—N1	104.99 (3)	C13—C14—H14	119.7
O4—S1—C11	108.96 (3)	C14—C15—H15	120.1
O3—S1—C11	109.66 (3)	C16—C15—H15	120.1
N1—S1—C11	104.59 (3)	C11—C16—H16	120.5
C16—C11—C12	121.87 (6)	C15—C16—H16	120.5
C16—C11—S1	119.63 (4)	C23—C22—H22	119.9
C12—C11—S1	118.47 (5)	C21—C22—H22	119.9
C13—C12—C11	118.58 (6)	C22—C23—H23	119.5
C12—C13—C14	120.13 (6)	C24—C23—H23	119.5
C15—C14—C13	120.64 (6)	C26—C25—H25	119.6
C14—C15—C16	119.80 (6)	C24—C25—H25	119.6
C11—C16—C15	118.98 (5)	C25—C26—H26	119.9
C26—C21—C22	119.33 (5)	C21—C26—H26	119.9
C26—C21—C7	122.53 (5)	C24—C27—H27A	109.5
C22—C21—C7	118.14 (5)	C24—C27—H27B	109.5
C23—C22—C21	120.19 (5)	H27A—C27—H27B	109.5
C22—C23—C24	120.92 (6)	C24—C27—H27C	109.5
C23—C24—C25	118.58 (5)	H27A—C27—H27C	109.5
C23—C24—C27	120.83 (6)	H27B—C27—H27C	109.5
			
C5—N1—N2—C3	3.82 (6)	O4—S1—C11—C12	178.56 (5)
S1—N1—N2—C3	158.10 (4)	O3—S1—C11—C12	45.42 (6)
N1—N2—C3—O1	175.68 (5)	N1—S1—C11—C12	−66.72 (5)
N1—N2—C3—C4	−2.54 (6)	C16—C11—C12—C13	−0.29 (10)
N2—C3—C4—C5	0.39 (7)	S1—C11—C12—C13	177.93 (6)
O1—C3—C4—C5	−177.85 (5)	C11—C12—C13—C14	−0.16 (12)
C3—C4—C5—N3	177.84 (6)	C12—C13—C14—C15	0.39 (12)
C3—C4—C5—N1	2.01 (6)	C13—C14—C15—C16	−0.17 (10)
N2—N1—C5—N3	−179.96 (6)	C12—C11—C16—C15	0.50 (9)
S1—N1—C5—N3	29.66 (9)	S1—C11—C16—C15	−177.70 (5)
N2—N1—C5—C4	−3.74 (6)	C14—C15—C16—C11	−0.26 (9)
S1—N1—C5—C4	−154.13 (4)	O2—C7—C21—C26	−172.68 (6)
O1—C6—C7—O2	−0.23 (7)	C6—C7—C21—C26	7.36 (8)
O1—C6—C7—C21	179.72 (5)	O2—C7—C21—C22	6.85 (8)
N2—C3—O1—C6	−3.11 (8)	C6—C7—C21—C22	−173.11 (5)
C4—C3—O1—C6	174.99 (5)	C26—C21—C22—C23	0.28 (9)
C7—C6—O1—C3	−173.56 (5)	C7—C21—C22—C23	−179.27 (5)
C5—N1—S1—O4	−159.90 (5)	C21—C22—C23—C24	0.51 (9)
N2—N1—S1—O4	50.57 (5)	C22—C23—C24—C25	−0.66 (9)
C5—N1—S1—O3	−31.07 (6)	C22—C23—C24—C27	179.40 (6)
N2—N1—S1—O3	179.40 (4)	C23—C24—C25—C26	0.02 (9)
C5—N1—S1—C11	84.36 (5)	C27—C24—C25—C26	179.96 (6)
N2—N1—S1—C11	−65.16 (4)	C24—C25—C26—C21	0.76 (9)
O4—S1—C11—C16	−3.18 (6)	C22—C21—C26—C25	−0.91 (9)
O3—S1—C11—C16	−136.32 (5)	C7—C21—C26—C25	178.62 (6)
N1—S1—C11—C16	111.54 (5)		

### Hydrogen-bond geometry (Å, º)

**Table:**

*D*—H···*A*	*D*—H	H···*A*	*D*···*A*	*D*—H···*A*
N3—H01···O1^i^	0.869 (13)	2.497 (14)	3.0124 (7)	118.7 (11)
N3—H01···O2^i^	0.869 (13)	2.048 (13)	2.9121 (7)	172.8 (13)
N3—H02···O3	0.863 (12)	2.121 (13)	2.7806 (8)	132.8 (11)
C14—H14···O2^ii^	0.95	2.54	3.3458 (8)	142

Symmetry codes: (i) −*x*, *y*+1/2, −*z*+1/2; (ii) *x*, −*y*+1/2, *z*+1/2.
